# An α-Smooth Muscle Actin (acta2/αsma) Zebrafish Transgenic Line Marking Vascular Mural Cells and Visceral Smooth Muscle Cells

**DOI:** 10.1371/journal.pone.0090590

**Published:** 2014-03-03

**Authors:** Thomas R. Whitesell, Regan M. Kennedy, Alyson D. Carter, Evvi-Lynn Rollins, Sonja Georgijevic, Massimo M. Santoro, Sarah J. Childs

**Affiliations:** 1 Department of Biochemistry and Molecular Biology, and Smooth Muscle Research Group, University of Calgary, Calgary, Alberta, Canada; 2 VIB Vesalius Research Center, University of Leuven (KU Leuven), Leuven, Belgium; University of Queensland, Australia

## Abstract

Mural cells of the vascular system include vascular smooth muscle cells (SMCs) and pericytes whose role is to stabilize and/or provide contractility to blood vessels. One of the earliest markers of mural cell development in vertebrates is *α smooth muscle actin (acta2; αsma)*, which is expressed by pericytes and SMCs. In vivo models of vascular mural cell development in zebrafish are currently lacking, therefore we developed two transgenic zebrafish lines driving expression of GFP or mCherry in *acta2*-expressing cells. These transgenic fish were used to trace the live development of mural cells in embryonic and larval transgenic zebrafish. acta2:EGFP transgenic animals show expression that largely mirrors native *acta2* expression, with early pan-muscle expression starting at 24 hpf in the heart muscle, followed by skeletal and visceral muscle. At 3.5 dpf, expression in the bulbus arteriosus and ventral aorta marks the first expression in vascular smooth muscle. Over the next 10 days of development, the number of acta2:EGFP positive cells and the number of types of blood vessels associated with mural cells increases. Interestingly, the mural cells are not motile and remain in the same position once they express the acta2:EGFP transgene. Taken together, our data suggests that zebrafish mural cells develop relatively late, and have little mobility once they associate with vessels.

## Introduction

New blood vessels form during angiogenesis from angioblasts that migrate into position and differentiate into endothelial cells. These ‘naked’ endothelial tubes then undergo a maturation process. In the next stage of angiogenesis, endothelial cells attract perivascular mural cells including pericytes found on smaller vessels, and smooth muscle cells (SMCs) found on larger vessels. The role of the mural cells is to physically support vessels, secrete extracellular matrix, provide vascular tone and induce vessel quiescence [1].

Hemorrhage results from breakage of contacts between endothelial cells, and can be due to a variety of mechanisms, either poor junctional contacts, defective extracellular matrix contacts, or lack the association of mural cells with endothelial cells [1], [2]. Reciprocal signalling events between endothelium and mural cells are critical for the maturation and stabilization of new vessels [3]. Endothelial cells express the chemoattractant PDGF-B, to attract mesenchymal cells expressing the PDGFRβ receptor to vessels [4]. In turn, these mesenchymal cells secrete Angiopoietin1 [5], which binds to Tie2 receptors expressed on endothelial cells and promote their differentiation [6]. The mutual attraction of the mesenchymal and endothelial cells results in the two layers forming close contacts, followed by maturation of the mesenchymal cells into smooth muscle or pericyte cells. In addition, both pericytes and SMCs require Sonic hedgehog signalling (Shh) for normal vascular development [7],and for the induction of Angiopoietin1 expression [8], [9]. The requirement for Shh extends throughout the lifetime for some SMCs as it is indispensable for their survival [10], [11]. Finally, signalling through Notch3 and Sphingosine1 phosphate pathways promotes the investment of mural cells on endothelial tubes [12], [13].

In the head, pericytes and vascular SMCs derive from the ectomesenchymal lineage of the cranial neural crest (CNC), at least in the chick and mouse [14], [15].The ectomesenchymal lineage also produces cartilage and bone of the face, mesenchyme and some cells of the heart [16]. These neural crest cells migrate ventrally from the hindbrain rhombomeres to populate the region around the eye and around the pharyngeal arches, arriving by 24 hpf in the zebrafish [16]. FoxD3, TFAP2 and Sox10 are three genes that promote specification of neural and neural crest pigment derivatives, and repress ectomesenchymal fates [15], [17], [18]. However transcription factors that actively specify SMCs from ectomesenchymal cells are currently unknown.

Vascular SMCs (vSMCs) are found in large blood vessels where a continuous single or multilamellar SMC layer surrounds the endothelial cell lining and provides contractility to modulate blood flow and stability. SMCs are separated from the endothelium by a basement membrane. SMCs secrete a large amount of the blood vessel ECM, consisting mainly of Laminin, Collagen IV, Nidogen, Perlecan, and Fibulins. Secretion of ECM from vSMCs is vital, as loss of the collagen Col4a1 leads to perinatal hemorrhage [19], while loss of Fibulin4 leads to aneurysms [20], [21].

Pericytes are mural cells found in microvessels (smaller arterioles and capillaries), particularly in the brain, eye and kidney [22]. Unlike SMCs, pericytes do not form a continuous layer and are present as isolated cells. Pericytes are embedded within the basement membrane. Recent findings show that they also provide contractility to blood vessels [23]. Interestingly once pericytes cover vessels, this halts vascular remodelling and prevents further proliferation of endothelial cells [24], [25]. Pericytes thus control fundamental behaviours of endothelial cells.

Although there are suggestions that the lineage of pericytes and smooth muscle cells might be identical, and they express overlapping sets of molecular markers [3], the two cell types are defined as morphologically distinct. One of the main criteria used to distinguish pericytes and SMCs is whether the mural cell lies within or outside of the basement membrane [3]. In embryonic development, however, this cannot always be applied as the basement membrane is not always present during angiogenesis. For instance, in early zebrafish vessels, ultrastructure shows no evidence of a basement membrane, nor convincing expression of pericyte or smooth muscle specific molecular markers [26]. In this case, mural cells have been referred to as ‘mesenchymal’ or ‘perivascular support cells’ until they can be properly identified [8], [26].

We and others have developed markers for the early vascular mural cell lineage in zebrafish, and have shown that these markers are expressed at much later equivalent developmental stages than in other organisms such as the mouse [27], [28], [29]. Another early smooth muscle marker, transgelin (tagln) is first visualized by antibody around 80 hpf in zebrafish [30]. An tagln/sm22α-b transgene can also be seen in the ventral head vessels on the late third and fourth day of development [28]. This timing suggests that mural cells are developing concomitantly with angiogenesis, however, these cells are difficult to visualize without molecular markers. Miano et al. used electron microscopy to conclude that undifferentiated mural cells were in place around the dorsal aorta at 7 dpf, but could not likely be identified by histology [27]. Thus the development of molecular markers that can identify vascular mural cells in vivo in zebrafish is urgent.

In contrast, early visceral smooth muscle development has been well characterized in zebrafish. RNA for *smooth muscle myosin heavy chain* and *non-muscle myosin heavy chain-b* begins to be expressed around 50 hpf [29], [31]. We previously showed that mRNA for the early smooth muscle marker *tagln/sm22α-b* turns on at 56 hpf and *acta2* turns on at 60 hpf in the gut [29]. More mature smooth muscle markers such as *cpi17* and *smoothelin-b* turn on at 72 hpf. Transgenic zebrafish generated using the *tagln/sm22α-b* promoter highlight visceral smooth muscle development, but in these animals, vascular smooth muscle is difficult to visualize [28]. This useful animal model still lacks reagents for in vivo imaging which would highlight vascular mural cell developmental processes at the cellular level. Here we develop transgenic animals expressing GFP or mCherry under the mural cell promoter α-smooth muscle actin and trace the development of these vSMC in living embryos, showing that although vascular mural cells arise late in development, they form smooth muscle layers around blood vessels, and are associated with vascular stabilization.

## Materials and Methods

### Ethics statement

Zebrafish were maintained and staged as previously described [32]. All procedures in this study were specifically approved by the University of Calgary Animal Care Committee. Wild type Tupfel long fin (TL) zebrafish or Tg(6.5kdrl:mCherry)^ci5^, Tg(fli1a:EGFP)^y1^, Tg(fli1a:nEGFP)^y7^ were used for all experiments [33], [34], [35].

### Whole-mount in situ hybridization and immunostaining

Digoxigenin-labelled antisense RNA probes were used in whole-mount in situ hybridization as previously described, with the exception that embryos older than 7 days post- fertilization (dpf) were fixed in Dietrich's fixative [36]. The probe for *acta2* has been described [29]. For histological analysis, embryos were embedded in JB4 (Polysciences) and 7 µm sections were cut on a Leica microtome. For antibody staining, the Vectstain ABC Kit (Vector Labs) with mouse αGFP (JL-8, BD Clontech). For histology, sections were stained with hematoxylin and aqueous eosin. The transgelin rabbit polyclonal antibody has previously been described [30].

### Identification of Acta2 promotor and enhancer sequences

We used the Santa Cruz genome browser to identify regions of cross-species conservation focused on the proximal promoter and first intron of the mouse *acta2* which drives smooth muscle-specific expression [37]. We used available software prediction programs (PATCH1.0) from TRANSFAC [38] to predict one potential CArG site. Other CArG sites were identified by manual inspection using validated CArG sites from mice.

### Acta2 transgenic zebrafish

A 300 bp proximal promoter for *acta2* and 2165 bp fragment from the *acta2* intron 1 was cloned using the primers described [39]. The two genomic fragments were fused in a PCR reaction to make a 2465 bp enhancer/promoter construct (referred to as the *acta2* promoter from this point on) which was cloned into pDONRp4p1r and then using the three way Tol2 Gateway cloning system upstream of GFP or mCherry [40]. Both promoter-fluorophore constructs were then isolated from the Tol2 backbone by digestion with Xho I and Cla I before injection into early one stage embryos. All founders had similar expression patterns, and a single founder was chosen for further analysis (Tg(acta2:EGFP)^ca7^) or Tg(acta2:mCherry)^ca8^). We note that Tg(acta2:mCherry)^ca8^ embryos have substantially weaker fluorescence. Both transgenic lines have been deposited to the Zebrafish International Resource Center with the Catalog IDs ZL4966 and ZL4967.

### Imaging

Sections were photographed using a Leica DMR microscope equipped with an Optronics Magnafire camera and Nomarski optics. Adult heart was photographed on a Zeiss Stemi SV11 microscope equipped with a Zeiss HR camera. For confocal microscopy, embryos were live imaged after mounting in low melt agarose on glass bottom dishes on a Zeiss LSM 510 Meta or Zeiss LSM 700 microscope. Slices were taken at intervals ranging from 1- 3 µm on a 10, 20 or 40× objective and subject to 2 times averaging. Image stacks were processed using a Kalman stack filter in Image J or in Zen Blue and are presented as maximal intensity projections. For timelapse imaging, embryos were mounted in low melt agarose in a heated chamber and imaged at 60 minute intervals.

### Morpholino knockdown

FoxD3 MO1 (5′ tgctgctggagcaacccaaggtaag 3′) and Tfap2a 5.1 MO (5′ cctccattcttagatttggccctat 3′) published morpholinos [18] were obtained from Gene Tools LLC and dissolved in water. 2.5 ng of morpholino was injected into 1–4 cell stage embryos.

## Results

### Creation of an Acta2 promoter/enhancer construct for in vivo expression

**Figure 1:**
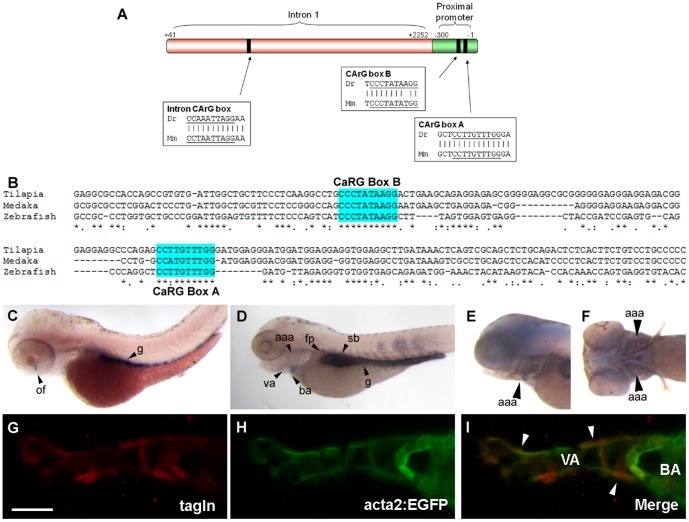
Acta2 promoter/enhancer construct design and expression in zebrafish. (A) A zebrafish (Dr) enhancer/promoter construct was constructed from the proximal promoter and first intron sequence of the zebrafish *acta2* gene, and contains three highly conserved CArG binding sites also found in the mouse (Mm) *acta2* proximal promoter and first intron. (B) Comparison of zebrafish CaRG boxes A and B in zebrafish, tilapia and medaka. (C,D) By wholemount in situ hybridization, *acta2* shows strong expression in the gut (g) at 72 hpf (B), and expressed in the gut, swim bladder (sb), ventral aorta (va), floor plate (fp), aortic arch arteries (aaa), and bulbus arteriosus (ba) at 100 hpf (C). (E,F) Co-localization of wholemount in situ hybridization *acta2* and anti-GFP staining of the acta2:GFP transgene shows strong expression in the aortic arch arteries (aaa) at 100 hpf. (G,H,I) 4 dpf acta2:EGFP transgenic fish (H) stained with Tagln rabbit polyclonal antibody (G). Merge (I) shows co-localization between acta2:GFP and Tagln. Arrowheads in G–I depict vascular mural cells. Scale bar in G represents 20 µm.

In mouse, both the proximal promoter and first intron of *acta2* contribute to its expression [37]. In particular ‘CArG box’ (CC (A/T)_6_ GG) motifs are critical for mural cell expression of *acta2*, including two CArG boxes in the *acta2* proximal promoter (CArG-A and CArG-B) and one in the first intron (intron CArG). We manually aligned the 300 bp proximal promoter and first intron of the zebrafish *acta2* gene with that of mouse. Although there is very poor sequence identity of these two genomic regions (42% overall identity), the relative position of the CArG elements is conserved (CArG-A is at -83 bp in fish and −70 in mouse; CArG-B is at −135 bp in fish and −121 in mouse; intron CArG is at +655 bp in fish and +1039 in mouse). Furthermore, the sequence of these elements is highly conserved from zebrafish to mouse, with only one conservative nucleotide difference in CArG-B, and absolute conservation in CArG-A and intron CArG (Figure 1A). In comparison with other fishes, an identical CArG-B box was also found in tilapia and medaka, as well as a completely conserved CArg-A box in tilapia (Figure 1B). These elements have not been functionally tested in zebrafish, although their conservation with those in mouse, tilapia and medaka, suggests they have been conserved through a long evolutionary period and are likely to be functionally important.

*acta2* is strongly expressed in visceral smooth muscle precursors in the gut and swim bladder at 72 hpf and 100 hpf (Figure 1C, D). Non-smooth muscle expression of *acta2* is also observed in the ventral eye in the site of the optic fissure (Figure 1C) and in the floor plate (Figure 1D).

### Acta2 is a marker of vascular mural cells and visceral smooth muscle

Hypothesizing that the first intron of *acta2* would act as a transcriptional enhancer in zebrafish as in mouse, we cloned the entire intron 1 upstream of 300 bp of proximal *acta2* promoter to make a compact promoter/enhancer construct that could easily be used for transgenesis (Figure 1A). The promoter/enhancer was adapted with Gateway cloning sites and inserted into the Tol2 transposon, driving either GFP or mCherry [40], [41]. To make transgenic animals, the promoter/enhancer:EGFP or promoter/enhancer:mCherry cassette was digested away from the transposon vector and the DNA injected into single cell embryos in a traditional transgenesis approach.

Multiple founders were identified from injection of the *acta2* promoter/enhancer construct. The alleles Tg(acta2:EGFP)^ca7^ and Tg(acta2:mCherry)^ca8^ were maintained for further study. Tg(acta2:EGFP)^ca7^ and Tg(acta2:mCherry)^ca8^ show identical expression patterns, although expression in Tg(acta2:mCherry)^ca8^ is weaker in intensity. For this reason, most experiments were conducted using Tg(acta2:EGFP)^ca7^ animals. We note that we have previously used the Tg(acta2:mCherry)^ca8^ line to demonstrate expression in the gut smooth muscle [39]. We also have previously used the Tg(acta2:GFP)^ca7^ line to show a decrease in vascular mural cells in the ventral head of βPix and integrin morphant animals [42] without characterizing the spatiotemporal expression of the transgene in the context of mural cell development.

To demonstrate the fidelity of the transgene, we compared transgene expression (as detected by an anti-GFP antibody) to that of native *acta2* transcript as detected by in situ hybridization. We find good concordance between both staining (Figure 1 E and F). We then compared acta2:EGFP expression in transgenic fish to native Tagln expression using antibody staining [30] (Figure 1 G–I), and find co-localization of Tagln and GFP.

**Figure 2:**
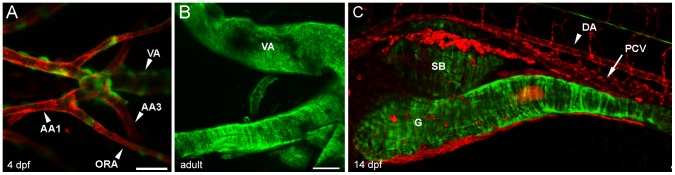
Morphology of vascular and visceral mural cells in acta2:EGFP transgenic fish. (A) Ventral pharyngeal region of a 4 dpf double transgenic Tg(acta2:EGFP)^ca7^; Tg(kdrl:mCherry)^ci5^ (mural cells are green and endothelial cells are red) zebrafish shows extensive mural cell coverage of the ventral aorta (VA) and lesser coverage on the smaller aortic arches (AA) or opercular artery (ORA). (B) Wholemount adult ventral aorta and attached afferent branchial arteries shows extensive smooth muscle coverage. (C) Lateral view of the gut (g) and swim bladder (b) of a 14 dpf double transgenic Tg(acta2:EGFP)^ca7^; Tg(kdrl:mCherry)^ci5^ zebrafish shows radial and circumferential smooth muscle on both gut and swim bladder, but sparse mural cells on the dorsal aorta (DA) and no visible cells on the posterior cardinal vein (PCV). Scale bar in A represents 25 µm. Scale bar in B and C represents 100 µm.

To demonstrate that smooth muscle was being labelled in the acta2:EGFP line, we first examined the morphology of cells expressing EGFP in vascular and visceral beds in double transgenic acta2:EGFP; kdrl:mCherry fish. In the pharyngeal region of 4 dpf embryos, the ventral aorta shows numerous acta2:EGFP cells exterior to the endothelial lining of the blood vessel, marked by Tg(kdrl:mCherry)^ci5^ shown in red (Figure 2A, Figure S1). Thus, acta2:EGFP cells meet the classic definition of ‘vascular mural cells' in being closely associated with the endothelial cell wall. As the coverage of the endothelium is not continuous at this stage of development, these cells are morphologically more similar to pericytes than smooth muscle cells. It is interesting that even though the ventral aorta is the vessel receiving the highest blood pressure in the zebrafish embryo, during embryonic development there is no clear evidence of a multilamellar vascular muscle wall as would be present in mammalian species at an equivalent developmental stage [28], [43]. To determine whether these pericyte-like cells eventually mature into smooth muscle, we dissected whole adult ventral aorta and associated vessels of an adult transgenic animal. We find that there is extensive coverage of the ventral aorta and associated vessels in the adult acta2:EGFP transgenic fish, suggesting that these early pericyte-like acta2:EGFP positive cells mature into a conventional smooth muscle layer as the fish grows (Figure 2B).

Clear evidence of radial and circumferential smooth muscle cells is seen in both the gut and the swim bladder of 14 dpf acta2:EGFP in the trunk region (Figure 2C). In contrast to the well-developed visceral smooth muscle, very few acta2:EGFP cells are seen on the dorsal aorta of the trunk, even at this juvenile stage. Skeletal muscle fiber expression of the acta2:EGFP transgene is present but highly variable from embryo to embryo at early stages (Figure S2).

### *acta2* expression in the developing heart outflow tract

**Figure 3:**
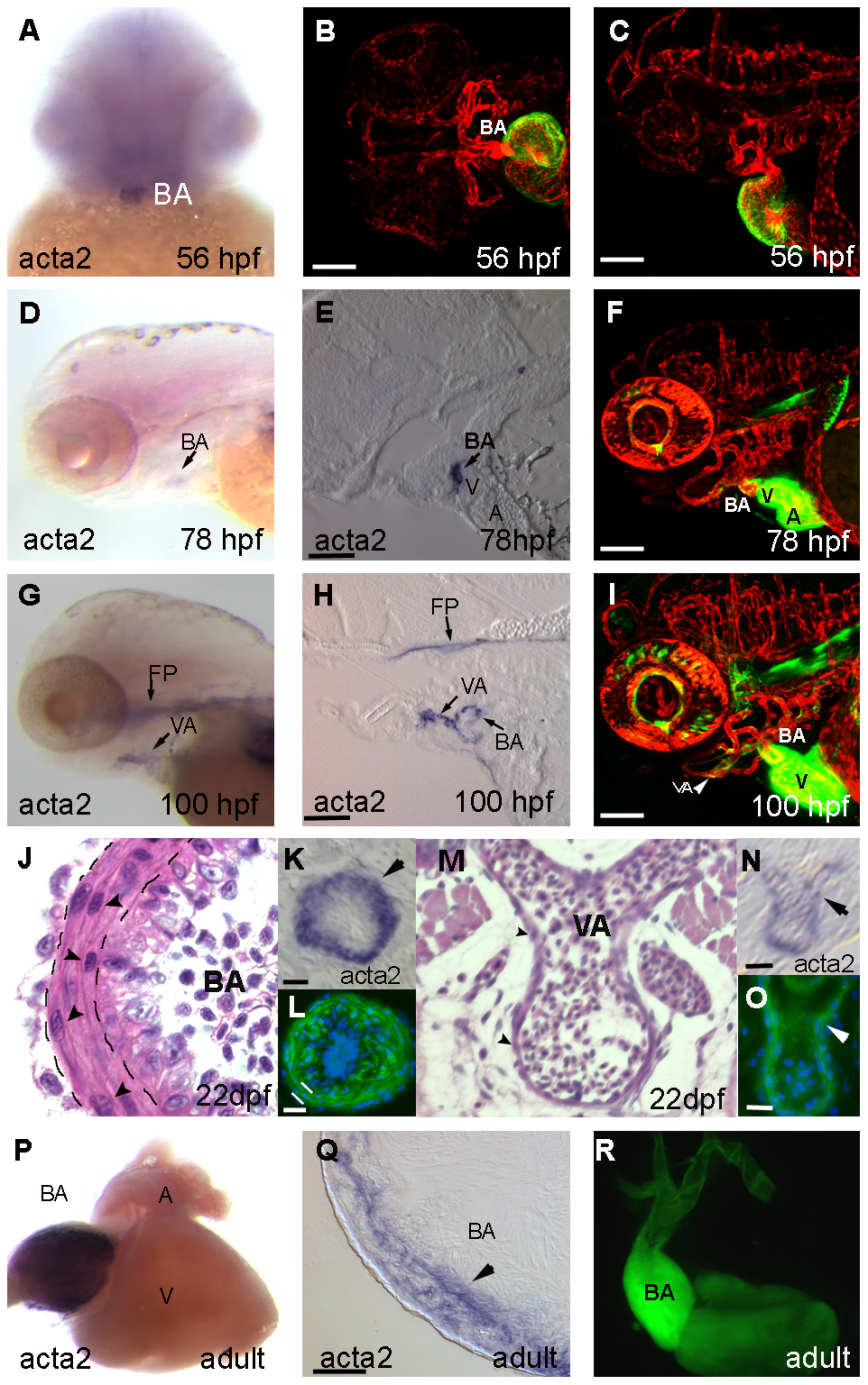
Smooth muscle markers are restricted to the developing cardiac outflow tract by 56 hpf. (A) At 56 hpf, *acta2* expression is restricted to the developing BA. (B,C) Double transgenic Tg(acta2:EGFP)^ca7^; Tg(kdrl:mCherry)^ci5^ embryo shows expression of EGFP in both the atrium and ventricle of the heart at 56 hpf, but not in the BA. (D) *acta2* expression is evident at 78 hpf in the BA in both wholemount and cross section (E) and in transgenic animals (F). (G–I) Expression of *acta2* continues to be restricted to the BA and ventral aorta (VA) at 100 hpf by in situ hybridization and in transgenic fish. (J–O): Cross sections of the 22 dpf BA show a multilamellar arterial phenotype as visualized by hematoxylin and eosin staining (J), in situ hybridization of *acta2* (K) and transgenic GFP (nuclei stained blue with DAPI, L). The bulbus vascular wall consists of three layers: an inner intima, middle media, and outer adventitia (Ad, separated by black lines in J). The intima is endothelial (arrowheads point to nuclei of endothelial cells). The media consists of 3–4 cell-thick layers of vascular smooth muscle cells (M, arrows point to nuclei of SMCs). In comparison to the BA, the vascular wall of the VA at 22 dpf is thin (M) but expresses *acta2* by in situ hybridization (N) and GFP in transgenic animals (O). The endothelium of VA is covered by a thin layer of SMCs (arrowheads point to nuclei of SMCs). (P) In situ hybridization of the wholemount adult heart shows strong staining in the bulbus arteriosus, but not ventricle or atrium, which is localized to the myocardial wall in cross section (Q). (R) Wholemount dissected acta2:EGFP transgenic heart shows stronger expression of GFP in the bulbus arteriosus as compared to ventricle. Staining is also continuous with the ventral aorta. In B,C, F, I, and R, green expression is acta2:EGFP transgene. Scale bar in B, C, F, and I is 100 µm. Scale bar in E, H, and Q is 50 µm. Scale bar in K, L, N, and O is 20 µm.

Since the region of the outflow tract has the highest blood pressure exposure, we hypothesized that it would be the first vessels to develop vascular smooth muscle. We examined native *acta2* transcript by in situ hybridization and compared it to acta2:EGFP transgene expression. At 56 hpf *acta2* mRNA is restricted to the bulbus arteriosus (Figure 3A). In contrast, the acta2:EGFP transgene is expressed in the myocardium of the atrium and ventricle, but not in the bulbus arteriosus at this time (Figure 3B,C). The lack of expression of the acta2:EGFP transgene in the bulbus arteriosus at 56 h hpf likely reflects a delay in GFP protein expression, as both *acta2* mRNA and acta2:EGFP transgene are expressed in the bulbus arteriosus at 78 hpf (Figure 3D–F). At 100 hpf, mRNA for *acta2* and the acta2:EGFP transgene are both visible in the bulbus arteriosus and ventral aorta (Figure 3G–I). We note persistent expression of the acta2:EGFP transgene in the atrium and ventricle at every time point examined including 78 and 100 hpf and beyond, times at which native *acta2* expression is not observed by in situ hybridization (Figure3 F, I). As myocardial expression continues into the adult, this suggests our *acta2* promoter-enhancer construct is lacking additional sequence to properly downregulate its expression in the heart. Additional non-muscle sites of *acta2* mRNA expression are seen in the tip of the notochord and floorplate (Figure 3 G–H). The anterior tip of the notochord is strongly labelled in acta2:EGFP transgenic fish (Figure 3F,I).

We next studied larval stages. At 22 dpf, multiple layers of smooth muscle are observed in the bulbus arteriosus by histology (Figure 3J), while the ventral aorta still has only a single layer (Figure 3M). This smooth muscle is positive for *acta2* expression by in situ hybridization (Figure 3K, N) and expresses the acta2:EGFP transgene (Figure 3L, O). Anatomical context for Figure 3 K and N is provided in Figure S3.

We then observed *acta2* expression in the adult heart. In situ hybridization of the wholemount heart shows strong staining of *acta2* in the bulbus arteriosus, but not atrium or ventricle (Figure 3P). Sections of the heart reveal that the *acta2* staining is localized to the muscle wall (Figure 3Q). In comparison, a whole heart isolated from an acta2:EGFP transgenic animal shows intense acta2:EGFP expression in the bulbus arteriosus, but maintains weak GFP expression in the ventricle (Figure 3R).

### acta2:GFP vascular mural cells gradually increase in number

**Figure 4:**
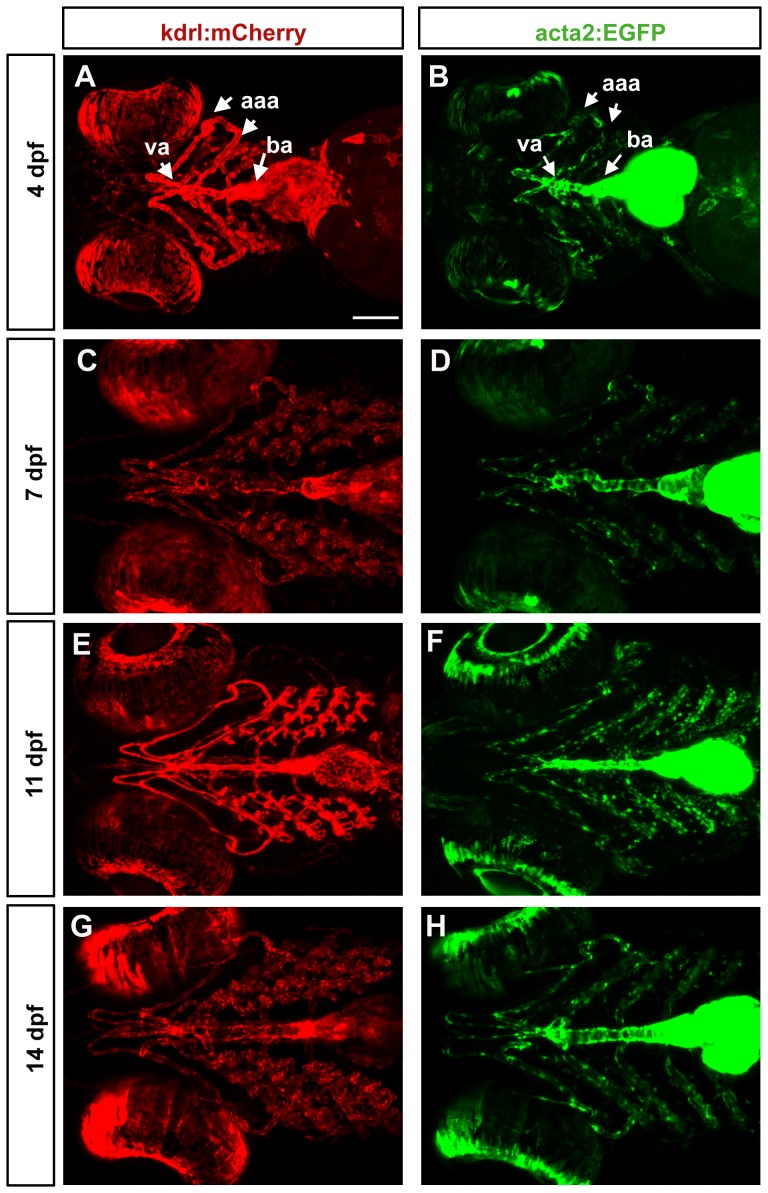
Mural cell and endothelial development in the ventral head of larval zebrafish. Confocal micrographs collected from a ventral point of view show a progressive increase in vessel complexity (red, A, C, E, G) and in density of mural cell coverage of aortic arch vessels (green, B, D, F, H) from 4 dpf (A, B), 7 dpf (C, D), 11 dpf (E, F) through 14 dpf (G, H). Heart expression of acta2:EGFP is maintained. aaa =  aortic arch arteries; va =  ventral aorta; ba =  bulbus arteriosus. Scale bar in A represents 100 µm.

To examine the progression of mural cell association with endothelium, we imaged acta2:EGFP cells in the ventral head at 4, 7, 11 and 14 days post fertilization. Zebrafish grow at variable rates during larval periods [44], and thus we imaged a minimum of 3 fish for each time point and present a representative image. The location of acta2:EGFP cells is compared to the pattern of blood vessels as marked by the Tg(kdrl:mcherry)^ci5^ transgene. At 4 dpf in ventral views (Figure 4), both chambers of the heart are strongly acta2:EGFP positive, as is the bulbus arteriosus. Along the ventral aorta, coverage by acta2:EGFP cells is dense, but does not completely cover the blood vessel. Scattered cells are seen on aortic arch arteries. At 7, 11 and 14 dpf the ventral aorta is still undergoing morphogenesis and increases in length. During this period, the coverage of acta2:EGFP cells increases modestly. Over this period we observe an increasing complexity in vascular pattern of the gill arches, although the majority of acta2:EGFP positive cells are associated with larger vessels and not newly formed small vessels.

**Figure 5:**
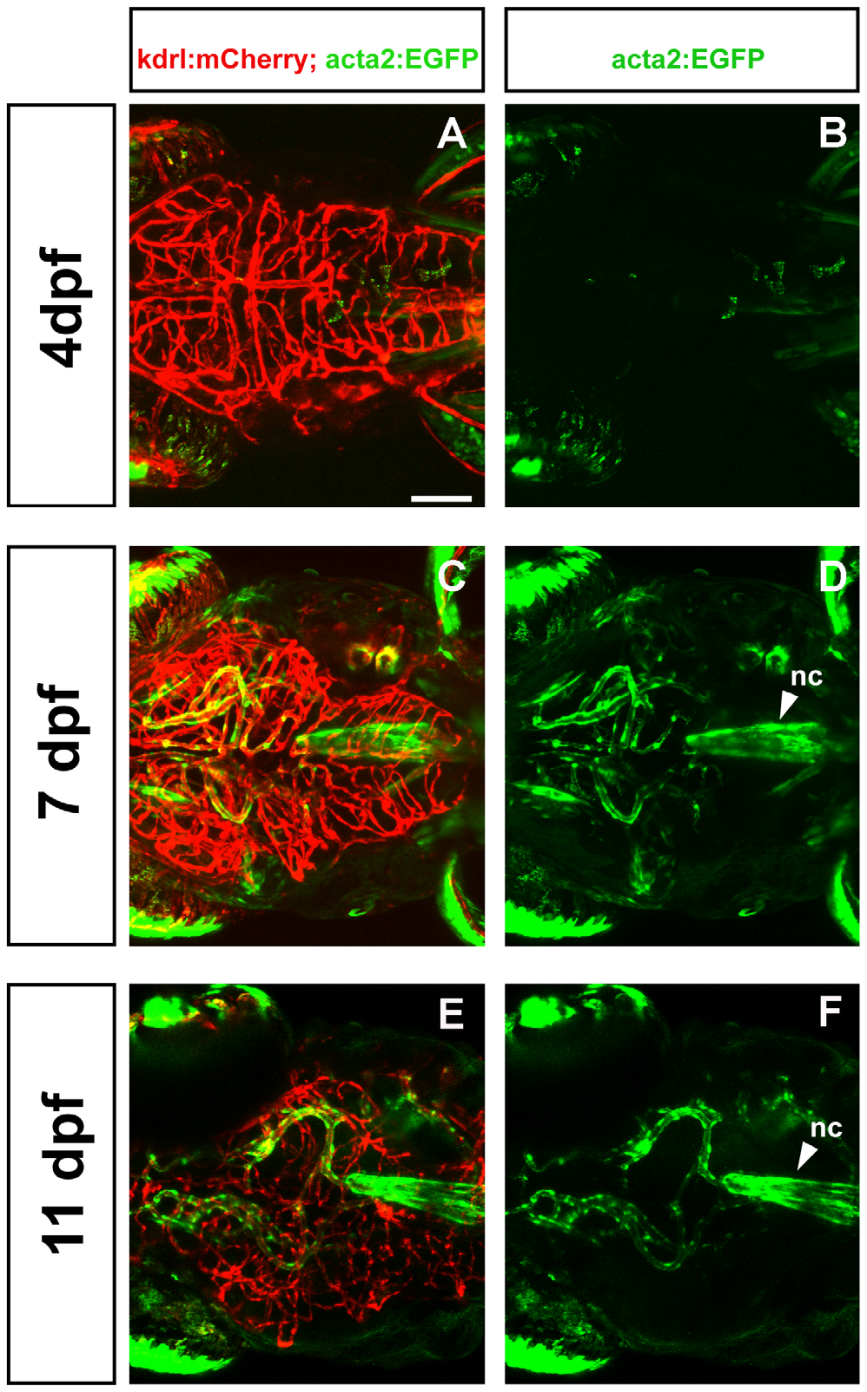
Development of mural cells and endothelial cells as seen in dorsal view. Confocal micrographs collected from a dorsal point of view show a progressive increase in vessel complexity (red, A, C, E) and in density of mural cell coverage of head vessels (green, B, D, F) at 4 dpf (A, B), 7 dpf (C, D), and 11 dpf (E, F). nc =  notochord. Scale bar in A represents 100 µm.

In dorsal view at 4 dpf only a few acta2:EGFP cells are observed (Figure 5B,D,F) despite extensive vascularization of the brain (Figure 5A,C,E). At 7 dpf and 11 dpf large head vessels are associated with acta2:EGFP cells.

**Figure 6:**
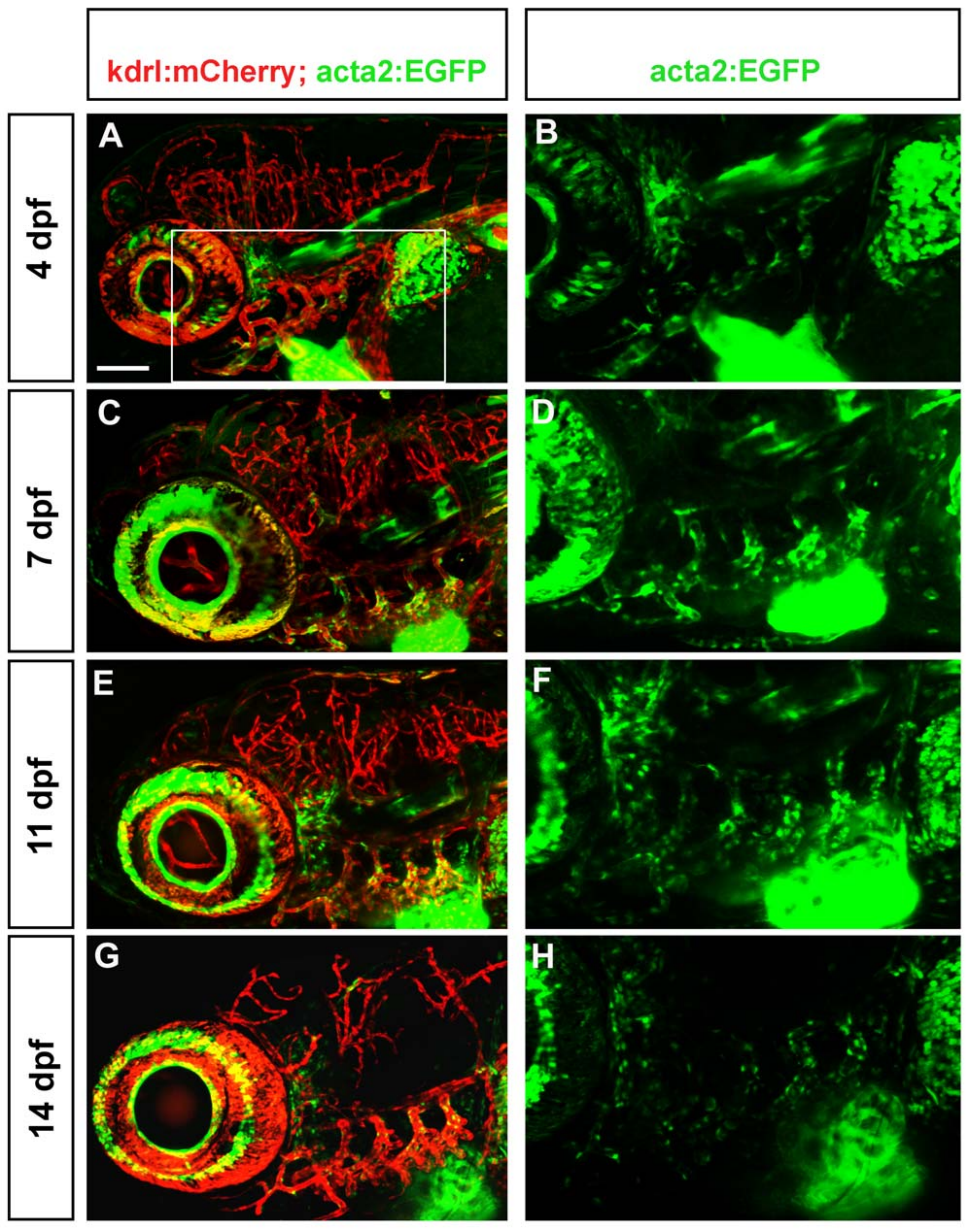
Development of mural cells and endothelial cells as seen in lateral view. Confocal micrographs collected from a lateral point of view show a progressive increase in vessel complexity (red, A, C, E, G) and in density of mural cell coverage of aortic arch vessels (green, all panels, inset is enlarged in B, D, F, H to show coverage of aortic arches) at 4 dpf (A, B), 7 dpf (C, D), and 11 dpf (E, F). Scale bar in A represents 100 µm.

In lateral view, we observe a striking scarcity of acta2:EGFP cells in the brain at early stages from 4 dpf to 14 dpf (Figure 6A,C,E,G). However there is extensive association of acta2:EGFP cells with ventral aortic arch vessels with the coverage becoming more complete over time (Figure 6B,D,F,H).

### Vascular trunk acta2:EGFP positive vascular mural cells are scarce

**Figure 7:**
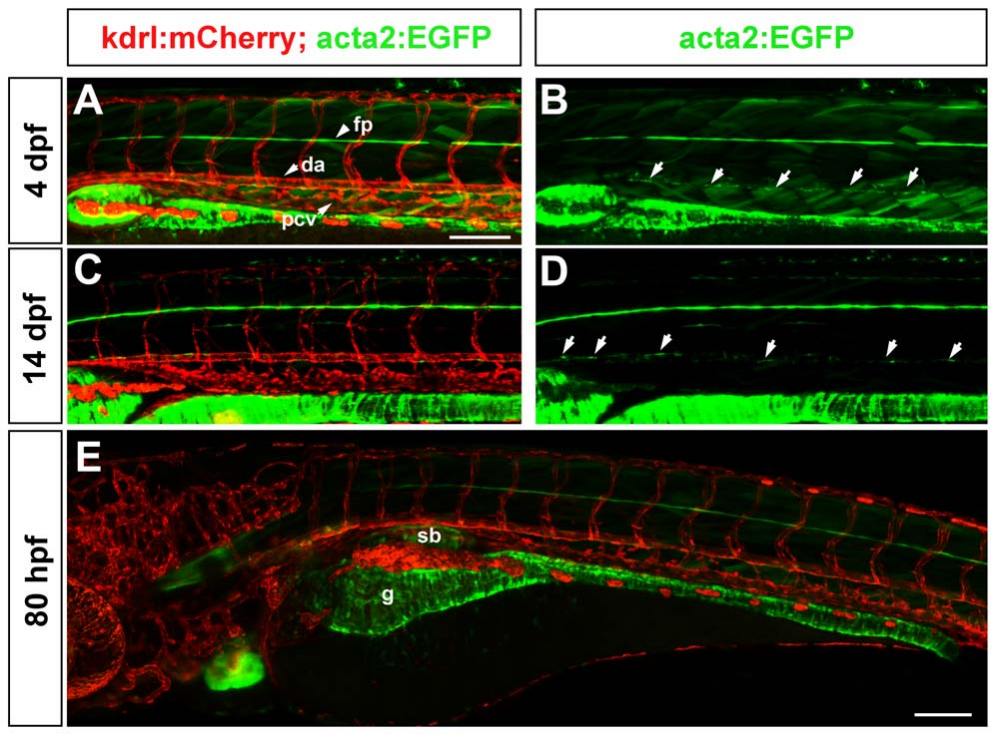
Vascular and visceral mural and smooth muscle cells in the trunk. (A–B) At 4 dpf, acta2:EGFP positive cells (arrows in B) are seen in the ventral portion of the dorsal aorta, but not in other vessels of the trunk. Floor plate (fp) expression of acta2:EGFP is observed in all images. (C–D) At 14 dpf, the distribution of vascular mural cells to the ventral portion of the dorsal aorta only, is still observed. (E) In contrast to the scarce vascular smooth muscle coverage, visceral smooth muscle cells strongly express the acta2:EGFP transgene at 80 hpf. Scale bars represent 100 µm. Green striations are skeletal muscle fibres.

In the trunk, vascular acta2:EGFP cells are scarce and are associated only with the ventral surface of the dorsal aorta at 4 dpf (Figure 7A,B). At this stage, these mural cells do not encircle the vessel however they are located outside the endothelium (Figure S1). At 14 dpf the morphology of the acta2:EGFP cells on the ventral aorta is similarly sparse, although a few acta2:EGFP cells can be seen on the ventral aspect of some intersegmental arteries suggesting that these angiogenic vessels are beginning to develop associations with mural cells (Figure 7C,D). In contrast, at 80 hpf, visceral smooth muscle is well developed and strongly expresses acta2:EGFP (Figure 7E). Variable, scattered skeletal muscle fibres express the acta2:EGFP transgene, although this diminishes over time.

### Embryonic origins of acta2:EGFP vascular mural cells

**Figure 8:**
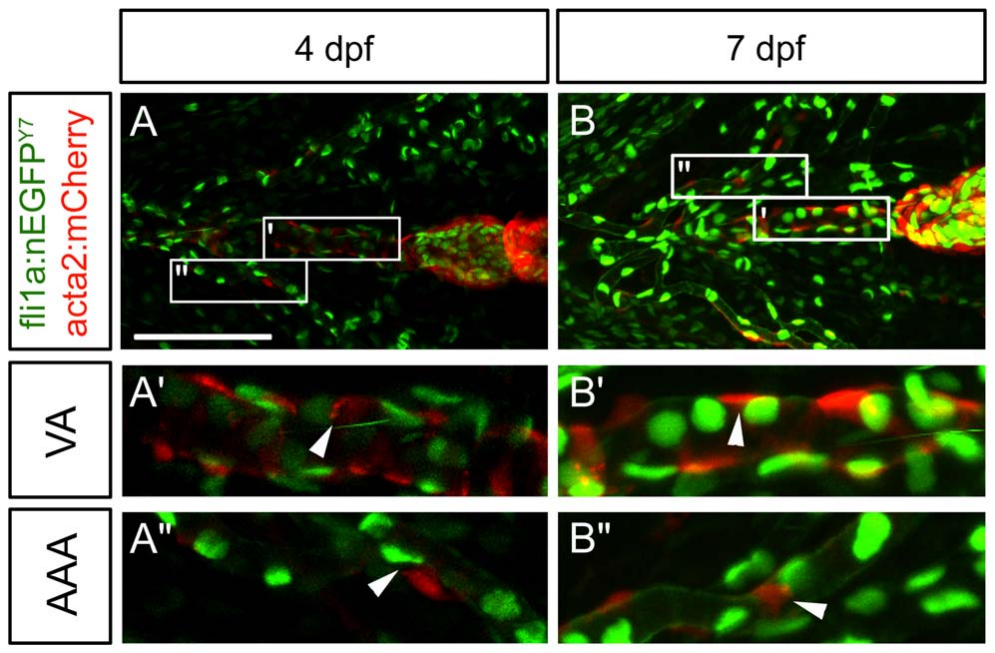
Lack of co-localization of mural cell and the ectomesenchymal neural crest marker Fli1a. Confocal images of 4 and 7(acta2:mCherry) and nuclear neural crest marker (fli1a:nEGFP^y7^) using ventrally staged embryos. (A) Mural cell and neural crest markers are expressed along the ventral aorta (A’) and aortic arch artery region (A”) of the 4 dpf embryo. (B) Mural cell and neural crest markers are expressed along the ventral aorta (B’) and aortic arch region (B”) at 7 dpf. There appears to be little to no co-localization of fluorescent markers at both 4 and 7 dpf. Scale bar in A represents 100 µm. Insets (A’, A”, B’, B”) are 100 µm in length. VA =  Ventral Aorta, AAA =  Aortic Arch Arteries. Arrowheads depict cells that no do not co-localize.

In mouse, chicken, and frog, vascular mural cells of the head originate from a migratory population of neural crest cells [14], [45], [46], [47]. There is no information on the origins of zebrafish head mural cells, therefore we crossed our transgenic acta2:mCherry^ca8^ fish with transgenic fli1a:nEGFP^y7^ zebrafish. Fli1a:nEGFP^y7^ labels endothelial cells and ectomesenchymal neural crest derivatives of the ventral head but not mesodermal or endodermal derivatives [34], [48]. If mural cells derive from a neural crest lineage, we might expect co-localization of fli1a:EGFP and acta2:mCherry, however, at 4, 7, and 10 dpf (Fig 8A, 8B, data not shown, respectively), we do not see co-localization of markers. We observe mCherry positive mural cells in proximity with GFP positive endothelial cells, but no obvious co-localization. This includes cells on the ventral aorta (Fig 8A’ and B’), and the aortic arch arteries (Fig 8A’’ and B’’), which are some of the first vessels to be covered with mural cells. These experiments could suggest that vascular mural cells of the ventral head arise from a non-neural crest origin, however we cannot rule out that *fli1a* expression has been downregulated in this lineage, or that the cells are neural crest derived but the *fli1a* transgenic fish line do not have the correct promoter/enhancer elements to express GFP in these cells.

As an independent test of origin we also tried ablating neural crest specification by knockdown of the transcription factors FoxD3 and TFAP2 [18]. We observed severely decreased numbers of acta2:EGFP positive cells; however embryos with double knockdowns of these transcription factors also had severely disrupted ventral head and endothelial patterning and circulation was compromised (Figure S4). Single knockdown of either FoxD3 or TFAP2A resulted in atypical, but less severely affected vessel patterning, with reduced mural cell coverage. These experiments are therefore inconclusive as mural cell differentiation could have secondarily been affected by the lack of robust circulation.

### acta2:EGFP expressing mural cells are stably associated with blood vessels

**Figure 9:**
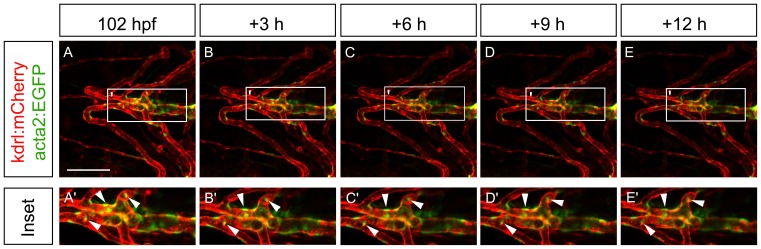
Vascular mural cells of the ventral head are very stable over time. (A–E) Single images taken from a confocal microscopy timelapse video. Images were collected at 102 hpf (A) and every three hours for 12 hours (B–E). Insets (A’–E’) show a higher magnification of the ventral aorta, where mural cells that are present at the beginning of the timelapse are still present at the end of the timelapse with no cytokinesis. Arrowheads depict mural cells throughout the timelapse that appear to have little movement. Scale bar represents 100 µm.

Data from in vitro models of endothelial-pericyte co-assembly suggests that pericytes are highly motile and migrate along nascent endothelial tubes in these culture systems [49]. We thus wanted to examine the behaviour of mural cells in vivo to determine their motility and proliferation when associated with vessels. We used a timelapse confocal microscopy strategy to follow acta2:GFP expressing cells on the ventral aorta and aortic arch vessels for several 12 hour windows from 3.5 through 5 days of development. In contrast to in vitro observations, we observe that cells expressing acta2:EGFP for the most part do not migrate, alter their cellular morphology or have observable cytokinesis during this window, and are therefore morphologically stable (Figure 9 A–E; Movie S1; n = 5 embryos). As the ventral aorta eventually becomes covered in multilamellar smooth muscle, we suggest that mural cells proliferate on very long time scale.

## Discussion

We show here that an acta2:EGFP transgenic line derived from an enhancer/promoter fusion is expressed in vascular mural cells and visceral smooth muscle cells in the zebrafish embryo, larva and adult. Visceral smooth muscle development has been well described by others using Tagln/SM22 transgenic zebrafish, and we will not discuss its formation further here as our observations in acta2:EGFP transgenic fish are similar [28]. Although *tagln* and *acta2* are both early smooth muscle markers with an essentially identical expression pattern in zebrafish [29], *acta2* turns on slightly later in development as detected by in situ hybridization. However, the temporal development of vascular smooth muscle as assayed by the Tagln/SM22 transgenic and our acta2:EGFP transgenic is similar. Seiler and Pack show expression of the Tagln/SM22 transgene at 4 dpf in the bulbus arteriosus and ventral aorta, similar to the acta2:EGFP transgenic [28]. However, the strong expression of acta2:EGFP in ca7 transgenic fish has allowed us to trace vascular mural cell development in live animals from late embryogenesis through larval and adult stages for the first time in this important model organism.

We demonstrate that vascular mural cells turn on acta2:EGFP several days after the initiation of circulation and initially show a pericyte-like morphology. Furthermore, it is only in late larval stages that multilamellar smooth muscle is observed. We corroborate transgenic expression with morphology and in situ hybridization for native acta2 mRNA. The most developed vascular smooth muscle occurs in bulbus arteriosus, with the next greatest coverage occurring in the ventral aorta. In contrast, the dorsal aorta of the trunk has single acta2:EGFP cells on the ventral surface only, at an equivalent time point. Hu et al., have shown that the ventral aorta has a 40% greater blood pressure than the dorsal aorta in the head of adult zebrafish due to the resistance in the fine branchial (gill) arteries [50]. Our observation of much greater mural cell coverage in the vessels adjacent to the heart (such as the ventral aorta) and much lower coverage in distal vessels (such as the dorsal aorta of the trunk) mirrors this blood pressure difference.

Why is there less vascular mural cell coverage of vessels in fish embryos as compared to mouse embryos? The reported ventricular systolic pressure in a 5 dpf zebrafish is 0.47 mmHg, while that of an adult is 2.5 mmHg [50], [51]. In comparison, the mouse blood pressure is around 2 mmHg at E9.5, but rises to 11.5 mmHg at E11.5 and 30 mmHg at P2[52]. Thus the blood pressure of a developing mouse is considerably higher than that of zebrafish. The need for vascular stabilization and control of blood vessel tone is clearly greater in a larger organism with much higher blood pressures [27]. Relatively low blood pressure may also explain why the first mural cells we observe have a morphology more similar to pericytes than smooth muscle as their function might be more important for vascular stabilization than in vascular tone.

Expression of *acta2* between mice and zebrafish also differs. Mice expressing a BAC-derived acta2:mCherry transgene show expression of acta2 at E8.5 in the myocardium of the heart [43]. At E9.5 and 10.5, signal is observable in the aorta and somites, while visceral smooth muscle expression begins at E13.5 in these mice. Thus mouse acta2 is expressed in vascular mural cells during embryonic stages concomitantly with the onset of circulation, while zebrafish *acta2* is expressed in larval and juvenile stages, long after the initiation of circulation. While the cell types that are labelled are similar between mouse and zebrafish, the order of their appearance differs. For instance, zebrafish show expression first in the myocardium, then in visceral smooth muscle and skeletal muscle before vascular smooth muscle expression. The slow development of vascular mural cells may be a reflection of the small size of the zebrafish and thus there is little need to develop contractile smooth muscle at an early stage. Unlike our zebrafish transgenic where acta2:EGFP is expressed in the myocardium through life, expression of the mouse acta2 transgene was maintained in adult smooth muscle, but not cardiac muscle.

acta2:EGFP expression seems to simply turn on in cells with a pericyte-like morphology as opposed to a gradual increase in intensity. We therefore predict there is an immature mural cell in place that is associated with vessels, which switches on acta2:EGFP expression when mature. We cannot visualize immature mural cells with our current transgenic model, or with any current marker. However we have previously shown the presence of vascular mural cells on the dorsal aorta of the hindbrain by transmission electron microscopy at 48–52 hpf. These cells lack contacts with endothelial cells in models of hemorrhage such as *igu* and *bbh* genetic mutants [8], [26]. Mural cells are therefore present and functional as early as 2 dpf, but do not express *acta2*, *tagln* or other early mural cell genes at this early stage. In the mouse, *acta2* and *tagln* are some of the earliest markers of the vascular mural cell lineage [53], but clearly, novel markers of even earlier mural cells are required in the zebrafish.

As head mural cells are thought to derive from neural crest, we wanted to test whether ectomesenchymal neural crest markers co-localized with the acta2 transgenic line. We show here that Tg(fli1a:nEGFP)^y7^, which is an ectomesenchymal neural crest marker, does not co-localize with acta2:mCherry. However, there are caveats to our double transgenic experiment because by the time that the acta2:mCherry transgene turns on at 4 dpf, fli1a:nEGFP may either never be expressed in vascular mural cells, or may be already downregulated. However, experiments to rule out that the vascular mural cells in the ventral head originate from lateral mesoderm are still required. This interesting question awaits a thorough lineage analysis.

We show that early vascular mural cells of the ventral aorta have the appearance of pericytes as they appear as single isolated cells on this vessel. Mural cells with a pericyte-like morphology can be found on large vessels in adult zebrafish. Some of these vessels would normally be covered in smooth muscle in other organisms. For instance, pericytes, not smooth muscle, are observed on adult coronary vessels [54]. Even the bulbus arteriosus, which is argued to be an enlarged artery, has been reported to have smooth muscle at 4 weeks, but not before [27], [55] although here we observe multilamellar smooth muscle at 22 dpf. As an aquatic organism with low blood pressure, more mural cell coverage may not be required.

The description of the acta2:EGFP transgenic line will now open the door to many unanswered questions in zebrafish vascular biology, allowing the simultaneous imaging of endothelial and mural cells during larval stages. This should allow us to address questions of origins, gene expression and behaviour of mural cells in this tractable model system.

## Supporting Information

Figure S1 **The acta2:GFP transgene is expressed surrounding endothelium in the ventral aorta.** Single slices of confocal micrograph stacks of double transgenic Tg(kdrl:mCherry; acta2:EGFP) zebrafish embryos at 7 dpf show green acta2:EGFP cells surrounding red kdrl:mCherry expressing endothelial cells in two different regions of the ventral aorta, distal (A) and proximate (B) to the heart outflow tract. The dorsal aorta is depicted in 11 dpf embryos, with green acta2:EGFP cells surrounding red kdrl:mCherry expressing endothelial cells (C) and with individual fluorescent markers (C’ - green acta2:EGFP cells; C” red kdrl:mCherry endothelial cells). Scale bar in B represents 20 µm, scale bar in C represents 50 µm.
(TIF)

Figure S2 **Wholemount image of 4 dpf acta2 transgenic zebrafish shows constant smooth muscle and heart expression and variable skeletal muscle expression.** Wholemount images of two independent 4 dpf zebrafish embryos using brightfield and fluorescent microscopy. While embryo 1 shows strong visceral smooth muscle expression and heart expression of the transgene, embryo 2 also shows scattered skeletal muscle fiber expression. The expression in skeletal muscle is variable from embryo to embryo and decreases over developmental time.
(TIF)

Figure S3 **In situ hybridization shows expression of acta2 in the Bulbus Arteriosus and Ventral Aorta.** Cross sections of 22 dpf zebrafish showing strong acta2 expression in the bulbus arteriosus and ventral aorta. This provides context to Figure 3 K and N. Scale bars are 50 µm.
(TIF)

Figure S4 **Single or double knockdown of FoxD3 or TFAP2a to block neural crest specification results in a reduction in acta2:GFP cells, but also severe ventral head and blood vessel patterning defects.** Representative brightfield images of 2 dpf zebrafish embryos show that both double knockdown (dMO) of FoxD3 and TFAP2A (C) or single knockdown (sMO) of FoxD3 (E) or TFAP2A (G), results in hemorrhage which is not present in control (A). Hydrocephalus of the hindbrain ventricle is also observed in dMO and sMO FoxD3. At 4 dpf, confocal microscopy shows that the control has a well-defined heart outflow tract, with mural cell coverage (kdrl:mCherry – red vessels; acta2:EGFP – green mural cells) (B). In dMO there are severe vessel malformations and a reduction in mural cell coverage (D). In the single FoxD3 (F) and TFAP2A (H) morphants, there are also malformations and reduced mural cell coverage, although these are less severe than the double morphant. Scale bar for A, C, E, G represents 200 µm. Scale bar for B, D, F, H represents 100 µm.
(TIF)

Movie S1 **Timelapse imaging of vascular mural cells reveals a stable phenotype over time.** Timelapse confocal microscopy of 102 hpf embryos (kdrl:mCherry – red vessels; acta2:EGFP – green mural cells) over a 12 hour timeframe, allowing for visualization of zebrafish embryo development. During this time period, mural cells do not appear to move or proliferate. Movie is representative of n = 5.
(AVI)
