# Supplementary figures and images for: An α-Smooth Muscle Actin (acta2/αsma) Zebrafish Transgenic Line Marking Vascular Mural Cells and Visceral Smooth Muscle Cells
Source: PLoS One. 2014 Mar 3;9(3):e90590. doi: 10.1371/journal.pone.0090590 (PMC3940907; doi:10.1371/journal.pone.0090590)

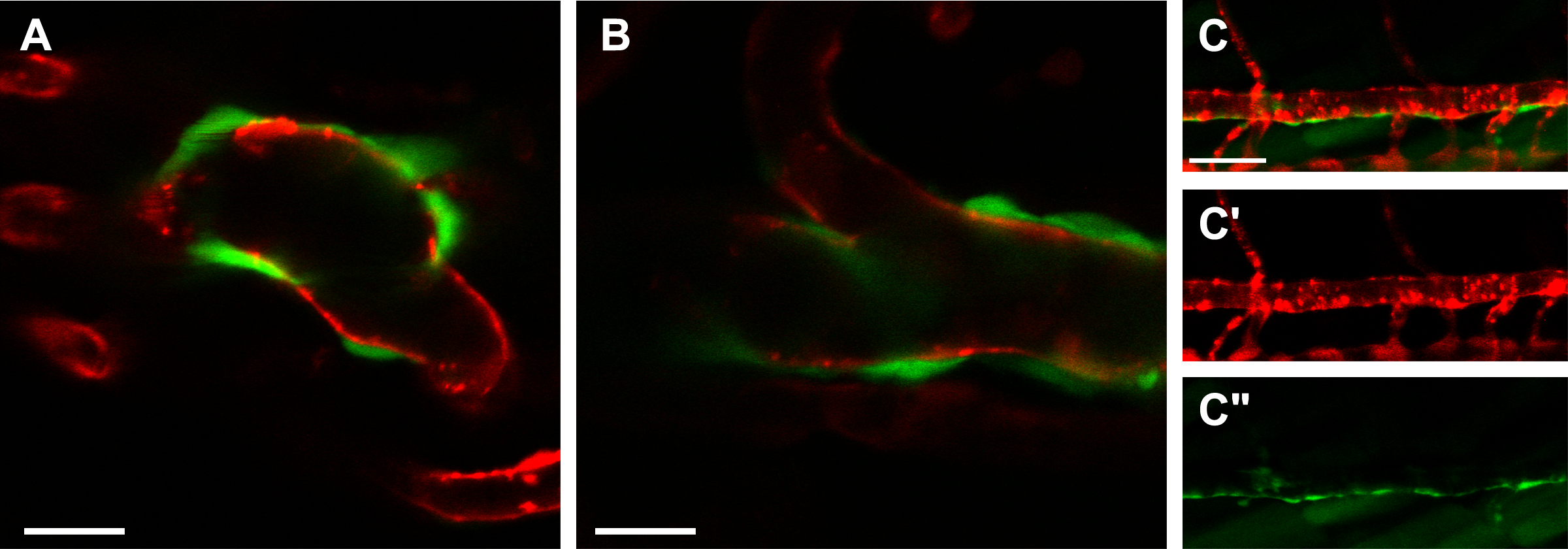

Supplement: Figure S1 — The acta2:GFP transgene is expressed surrounding endothelium in the ventral aorta. Single slices of confocal micrograph stacks of double transgenic Tg(kdrl:mCherry; acta2:EGFP) zebrafish embryos at 7 dpf show green acta2:EGFP cells surrounding red kdrl:mCherry expressing endothelial cells in two different regions of the ventral aorta, distal (A) and proximate (B) to the heart outflow tract. The dorsal aorta is depicted in 11 dpf embryos, with green acta2:EGFP cells surrounding red kdrl:mCherry expressing endothelial cells (C) and with individual fluorescent markers (C’ - green acta2:EGFP cells; C” red kdrl:mCherry endothelial cells). Scale bar in B represents 20 µm, scale bar in C represents 50 µm. (TIF) [file pone.0090590.s001.tif]

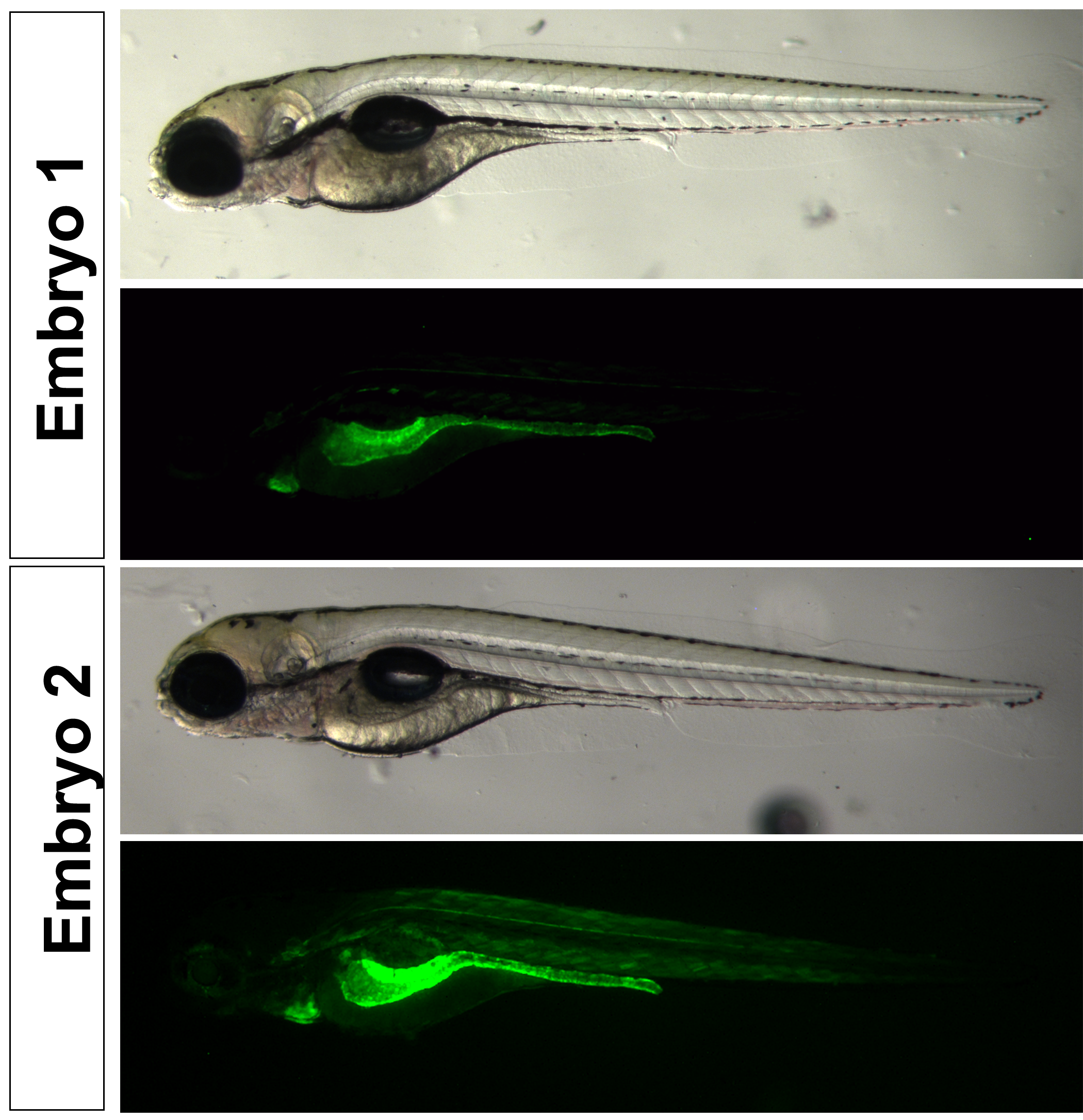

Supplement: Figure S2 — Wholemount image of 4 dpf acta2 transgenic zebrafish shows constant smooth muscle and heart expression and variable skeletal muscle expression. Wholemount images of two independent 4 dpf zebrafish embryos using brightfield and fluorescent microscopy. While embryo 1 shows strong visceral smooth muscle expression and heart expression of the transgene, embryo 2 also shows scattered skeletal muscle fiber expression. The expression in skeletal muscle is variable from embryo to embryo and decreases over developmental time. (TIF) [file pone.0090590.s002.tif]

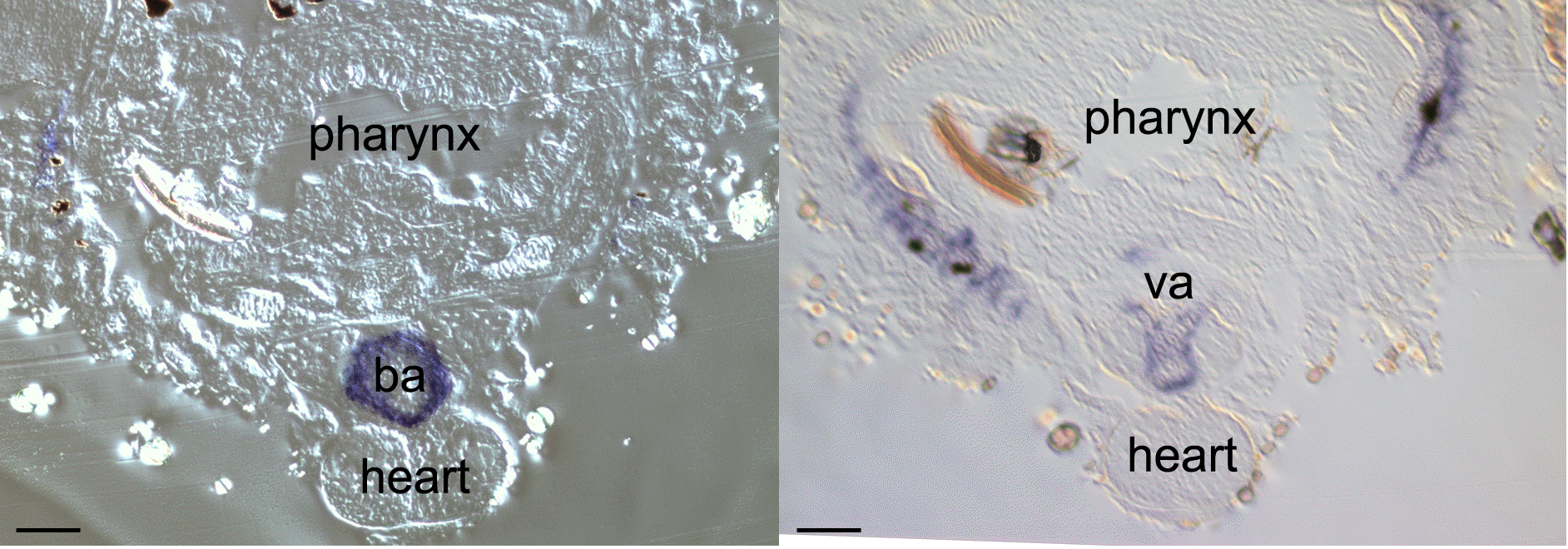

Supplement: Figure S3 — In situ hybridization shows expression of acta2 in the Bulbus Arteriosus and Ventral Aorta. Cross sections of 22 dpf zebrafish showing strong acta2 expression in the bulbus arteriosus and ventral aorta. This provides context to Figure 3 K and N. Scale bars are 50 µm. (TIF) [file pone.0090590.s003.tif]

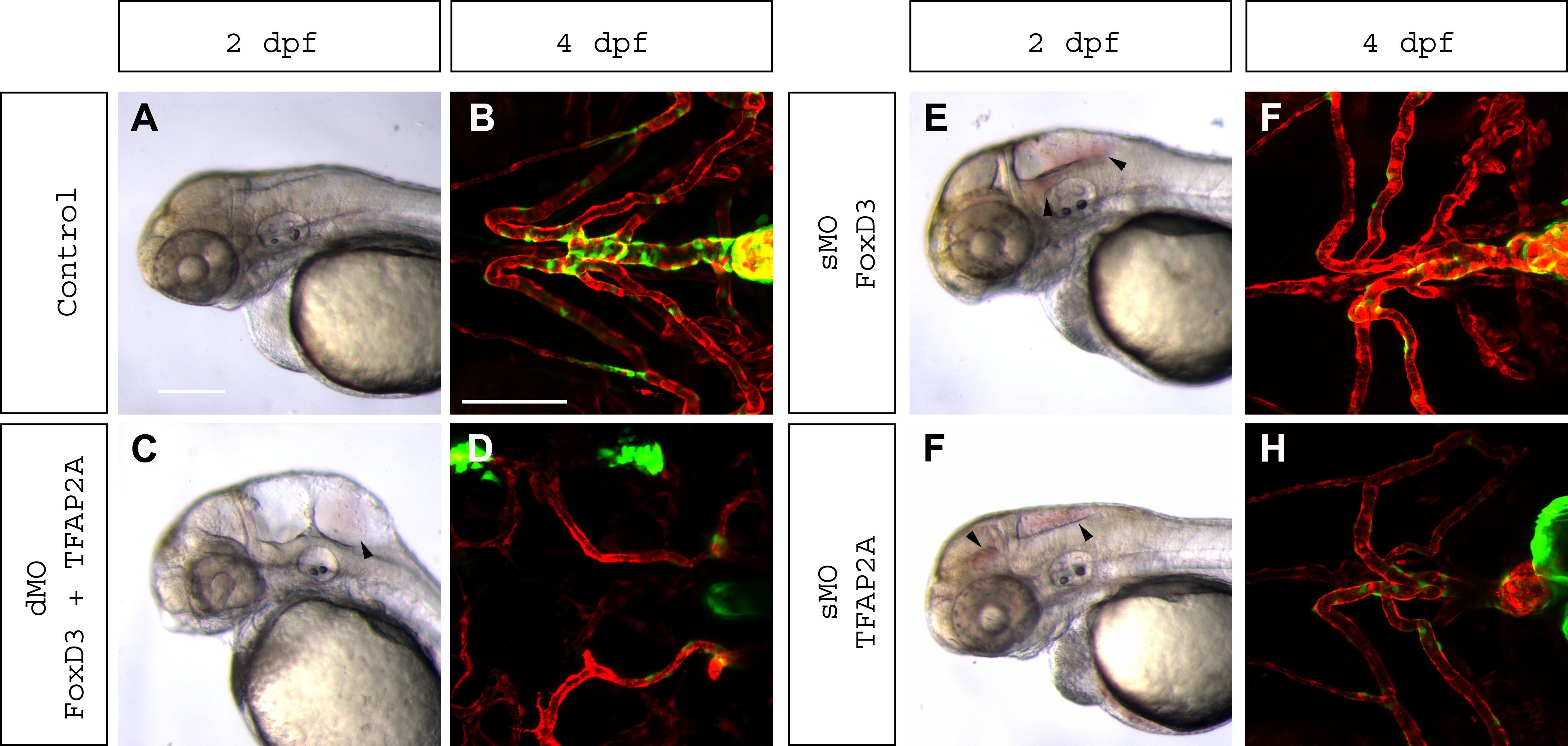

Supplement: Figure S4 — Single or double knockdown of FoxD3 or TFAP2a to block neural crest specification results in a reduction in acta2:GFP cells, but also severe ventral head and blood vessel patterning defects. Representative brightfield images of 2 dpf zebrafish embryos show that both double knockdown (dMO) of FoxD3 and TFAP2A (C) or single knockdown (sMO) of FoxD3 (E) or TFAP2A (G), results in hemorrhage which is not present in control (A). Hydrocephalus of the hindbrain ventricle is also observed in dMO and sMO FoxD3. At 4 dpf, confocal microscopy shows that the control has a well-defined heart outflow tract, with mural cell coverage (kdrl:mCherry – red vessels; acta2:EGFP – green mural cells) (B). In dMO there are severe vessel malformations and a reduction in mural cell coverage (D). In the single FoxD3 (F) and TFAP2A (H) morphants, there are also malformations and reduced mural cell coverage, although these are less severe than the double morphant. Scale bar for A, C, E, G represents 200 µm. Scale bar for B, D, F, H represents 100 µm. (TIF) [file pone.0090590.s004.tif]
